# Calcium Silicate Promoting the Upcycling Potential of Polysulfone Medical Waste in Load-Bearing Applications

**DOI:** 10.3390/jfb15110323

**Published:** 2024-10-30

**Authors:** Chi-Nan Chang, Jia-Jia Chung, Huei-Yu Jiang, Shinn-Jyh Ding

**Affiliations:** 1 Institute of Oral Science, Chung Shan Medical University, Taichung City 402, Taiwan; danycnc@gmail.com (C.-N.C.);; 2 School of Dentistry, Chung Shan Medical University, Taichung City 402, Taiwan; 3 Department of Stomatology, Chung Shan Medical University Hospital, Taichung City 402, Taiwan

**Keywords:** polysulfone, calcium silicate, upcycling, antibacterial activity, load bearing

## Abstract

Polysulfone (PSF) medical waste can be effectively repurposed due to its excellent mechanical properties. Due to the increasing need for load-bearing bone implants, it is crucial to prioritize the development of biocompatible polymer–matrix composites. Calcium silicate (CaSi), known for its osteogenesis and antibacterial properties, is widely used in medical applications. In this study, recycled PSF plastics in fiber or nanoparticle forms and commercial PSF products were used to create PSF-based composites filled with three different amounts (10, 20, and 30 vol%) of CaSi. The green compact was heat-treated at various temperatures. Experimental results showed that the mechanical interlocking of the PSF matrix and CaSi filler occurred due to the liquefaction of PSF fibers or nanoparticles during heat treatment. When the composite contained 20% CaSi, the obtained three-point bending strength exceeded 60 MPa, falling within the reported strength of compact bone. There was a concurrent improvement in the biocompatibility and antibacterial activity of the PSF-based composites with the increasing amount of CaSi. Considering their mechanical properties and antibacterial activity, the 20% CaSi-containing PSF-based composites treated at 240 °C emerged as a promising candidate for bone implant applications. This study demonstrated the feasibility of upcycling medical waste such as PSF as a matrix, opening doors for its potential usage in the medical field.

## 1. Introduction

Plastic waste has increased dramatically due to the widespread consumption of plastics globally and their low recycling rate. This has led to pervasive environmental pollution and severe waste of material resources [1,2]. If the reduction in and reuse of resources are not effectively carried out, recycling becomes even more critical. Proper recycling of resources and safe disposal of waste are crucial for ensuring environmental sustainability. It is concerning to note the wastage of expensive medical materials, especially when considering that Taiwan has the highest per capita population of over 90,000 people undergoing kidney dialysis globally [3]. Therefore, it is essential to recycle and remanufacture the primary polysulfone (PSF) polymer used in dialysis into valuable materials. This approach not only addresses the issues of air pollution and dioxin caused by the incineration of medical waste plastics but also helps in reducing landfill waste. PSF, as a high-performance transparent thermoplastic, offers notable advantages such as heat resistance, chemical stability, high permeability, biocompatibility, and X-Ray resistance [4,5]. Notably, it is the most widely used membrane material in hemodialysis and catheters [4,6]. PSF shows minimal complement activation, leukocyte reduction, and low release of leukocyte elastase [7,8]. Furthermore, its properties make it a promising material for bone implants due to its low density, non-toxic nature, and mechanical properties similar to bone tissue. For instance, Nechifor and colleagues have created PSF-silica microfiber grafted with TiO_2_ membranes for guided bone regeneration [9]. Developing high-value products from recycled medical waste is an intriguing area of study.

Metal materials like titanium and stainless steel are commonly used in artificial joints, dental implants, and bone fixation devices due to their exceptional mechanical properties. However, these metallic implants are susceptible to corrosion and the release of toxic elements when exposed to body fluids, leading to allergic reactions and inflammation [10]. Moreover, the high modulus of elasticity of metal implants (Ti: 120 GPa; stainless steel: 200 GPa) can cause bone atrophy due to stress shielding, as it surpasses that of cortical bone (7–30 GPa) [11,12]. In contrast to high-modulus metallic implants, various low-modulus polymeric materials, such as polyurethane (PU), polyethylene (PE), high-density polyethylene (HDPE), ultra-high-molecular-weight polyethylene (UHMWPE), and polyetheretherketone (PEEK), have been developed for bone repair and substitutes. For example, uniform incorporation of HA (hydroxyapatite) in a biodegradable polyurethane (PU) scaffold can stimulate bone repair in calvarial defects [13]. To address the issues under load-bearing conditions, there is an ongoing development of high-strength polymeric materials [13,14,15,16,17,18,19,20,21,22,23,24,25]. High-strength polymers, such as polylactic acid (PLA) and polyglycolic acid (PGA), have been engineered for bone implants, offering benefits such as lower modulus and density compared to metallic materials [13,14,15,16,17,18]. Additionally, polymer-based implants alleviate the stress-shielding effect, enable post-operative diagnostic imaging, and eradicate metal artifacts [16]. While absorbable PLA and PGA-based bone nails and plates can provide initial strength for facial fracture healing, potential side effects, such as the release of acidic substances, must be carefully considered [17,18].

On the other hand, biomedical advances in aging bone tissue defects and dental restoration demand biomaterials that offer long-term stability for successful clinical outcomes. To achieve this, the use of stable polymers such as PE [19,20], PEEK [21], and PSF [22,23], as well as methacrylate-based resins [16], in combination with bioceramics, has led to the creation of high-performance composites. These composites may demonstrate the potential to replace metal implants in orthopedic and dental applications. The ability to tailor physicochemical, biological, and mechanical properties for specific medical applications is a clear advantage of using polymer matrices and bioactive ceramics as bone implants [23,24,25]. For example, a bioactive, non-biodegradable HA/PEX (hydroxyapatite-reinforced PE composite) composite comprises a PE matrix reinforced with HA particles for bone augmentation [20]. Babar Munir’s group added ZnO and HA to the HDPE matrix to improve the mechanical and thermal properties of HDPE-based composites [26]. Carbon nanotubes (CNTs) and calcium silicate (CaSi) were added to PEEK to improve their mechanical properties and biological activities, respectively [27]. Laminated composites of HA/PSF exhibit improved fracture toughness over HA alone and similar mechanical properties to bone [28]. Researchers have even developed bioactive glass fiber/PSF composite implants for femoral hip prostheses to promote direct bone bonding [29]. Moving forward, further investigation is crucial to develop osteoconductive and antibacterial composites for their widespread clinical applications.

Bioactive CaSi has been shown to have antibacterial properties and promote stem cell growth, proliferation, and differentiation more effectively than materials [30,31,32]. Moreover, it exhibits outstanding remineralization ability, actively promotes bone regeneration [33,34], and takes advantage of the versatile capabilities of CaSi particles for drug delivery, bone filling, coating layer, injectable bone materials, and 3D scaffolds [35,36]. Its composites are considered effective therapeutic agents for the regeneration of hard and soft tissues [33,34,37]. Hence, a promising approach to producing the bioactive composites involved combining PSF with CaSi to promote osteogenesis. Cheng and Chang utilized a solvent casting–evaporation method to produce a PSF/CaSi film as a tissue barrier [38]. The significance of recycled PSF from dialysis tubes is noteworthy due to its excellent mechanical properties. Utilizing recycled PSF as a composite matrix not only helped reduce the cost of raw materials and prevented environmental contamination during incineration but also added value to the medical industry. This study delved into the potential of eco-friendly, recycled PSF-based composites for load-bearing applications. Additionally, there is a growing trend in developing antimicrobial biomaterials to tackle infection issues [39]. Non-antibiotic antimicrobial agents are crucial in addressing the challenges of drug-resistant bacterial infections [31,39]. This research was the first to combine high-strength recycled PSF with antibacterial CaSi for bone implants. Consequently, recycled PSF materials with fiber or nanoparticle types were employed to prepare high-strength and biocompatible composites by incorporating bioactive CaSi ceramics. The study investigated not only the phase and microstructure of the various PSF-based composites but also their mechanical properties, providing a comprehensive understanding of their characteristics. Furthermore, L929 cytotoxicity and the antibacterial efficacy against Gram-positive *Staphylococcus aureus* (*S. aureus*) and Gram-negative *Escherichia coli* (*E. coli*) bacteria were also evaluated, ensuring an assessment of the composites’ safety and effectiveness.

## 2. Materials and Methods

### 2.1. Preparation of Components

**Figure 1:**
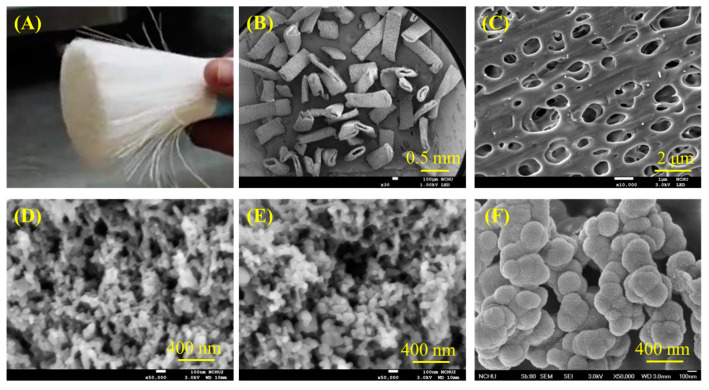
(**A**) Optical photograph of the recycled PSF fiber, SEM microscopic images of pieced PSF fiber at (**B**) low and (**C**) high magnification, (**D**) recycled PSF-derived nanoparticles, (**E**) commercial PSF-derived nanoparticles, and (**F**) CaSi particles.

The long hollow fiber PSF was sourced from Green Plastic Technology Corporation in Tainan, Taiwan (Figure 1A). These fibers were recycled from FX80 classix dialyzer by Fresenius Medical Care AG in Bad Homburg, Germany. Following autoclave sterilization, the recycled PSF fiber was cut into small pieces using the POWTEQ HM100 (Beijing Grinder Instrument, Beijing, China). The short fiber was sieved through a 270 μm mesh (no. 50). Additionally, commercial PSF pellets (MW = 35,000, lot no. 428302) were acquired from Sigma-Aldrich (St. Louis, MO, USA) for comparative purposes. Due to the challenges of utilizing the hard pellets of commercial PSF in preparing the composite, N-N-dimethylacetamide (99.5%, ECHO Chemicals, Miaoli, Taiwan) was used to dissolve Sigma-Aldrich PSF pellets at a ratio of 1/40 by weight to volume at 60 °C for 1 h under stirring, then cooled in ice water. After suction filtration to obtain the nanoparticles, the samples were dried at 60 °C. Conversely, recycled PSF fiber was also leveraged to prepare the PSF nanoparticles. For simplification, the recycled PSF fiber was referred to as RFB, while the nanoparticles prepared from the sources of recycled fibers and commercial pellets were named RNP and CNP, respectively.

Reagent-grade tetraethyl orthosilicate (Si(OC_2_H_5_)_4_, TEOS) from Sigma-Aldrich and calcium nitrate (Ca(NO_3_)_2_·4H_2_O) from Showa (Tokyo, Japan), were used as precursors for SiO_2_ and CaO, respectively. An alkaline cetyltrimethylammonium bromide (CTAB)-assisted precipitation method was utilized to prepare CaSi particles [35]. This involved adding Ca(NO_3_)_2_·4H_2_O to a CTAB (Sigma-Aldrich) solution in 2% ammonia solution (Wako, Osaka, Japan) for 1 h. Ethanol and TEOS were then sequentially introduced to the mixture. Following continuous stirring for 24 h, the mixture underwent centrifugation at 3500 rpm for 5 min, was washed twice with deionized water, and was then dried in an oven overnight at 120 °C. The resulting precipitated CaSi powders were then calcined in air at 800 °C, with a heating rate of 10 °C/min for 3 h to remove the surfactant template, and eventually cooled to room temperature.

### 2.2. Preparation of Composites

To prepare the PSF/CaSi composite, PSF was wet-blended with CaSi at different volume ratios (10:0, 9:1, 8:2, and 7:3) using a conditioning mixer (ARE-250, Thinky, Tokyo, Japan) for 10 min after adding ethanol and then dried in an oven at 60 °C for 1 day, which was modified from a previous study [40]. For simplicity, the samples were named RFB, RNP, and CNP, representing polymer from recycled fiber, recycled fiber precipitated into nanoparticles, and commercial pellet precipitated into nanoparticles, respectively, followed by a number indicating the volume ratio. For instance, “RFB91” designated a composite of recycled fibers containing 10 vol% CaSi particles. The green compact was obtained by molding the specimens within a stainless-steel mold under an applied pressure of 200 MPa (three-point bending and compressive strength) or 80 MPa (for tensile strength) for 1 min using a uniaxial press. This variation in applied pressure was due to the sample size and limitations of the hydraulic press. The green body was heat-treated at 220–260 °C at 20 °C intervals for 3 h at a 2 °C/min heating rate in the air to bind the PSF polymer and CaSi ceramic. The heat-treated composite was then cooled to room temperature.

### 2.3. Phase Composition and Surface Morphology

The phase analysis was carried out using X-Ray diffractometry (XRD; Bruker D8 SSS, Karlsruhe, Germany) with Ni-filtered Cukα radiation operated at 40 kV and 40 mA at a scanning speed of 1°/min. The chemical structure of specimens was examined using Fourier transform infrared spectroscopy (FTIR; Bruker Vertex 80v, Ettlingen, Germany) in a transmission mode within the wavenumber range of 400 to 4000 cm^−1^, with a spectral resolution of 1 cm^−1^, to measure the various functional groups. Optical images of the composites were captured to illustrate the change in shape before and after heat treatment using an iPhone 12. The surface morphologies were observed using a field-emission scanning electron microscope (SEM; JEOL JSM-7800F, Tokyo, Japan) after coating with gold using a JFC-1600 (JEOL) coater.

### 2.4. Measurement of Mechanical Properties

A specimen measuring 6 mm in diameter and 12 mm in length was utilized to assess its compressive properties in accordance with ASTM D695 standard. The compressive strength (CS) was determined using a static mechanical testing machine (AGS, Shimadzu, Kyoto, Japan) equipped with a 10 kN load cell at a crosshead speed of 1 mm/min. The calculation of the CS value for each specimen was based on the equation CS = P/πr2, where P is the peak load (Newtons, N) and r is the radius (mm) of the specimen. The maximum compression load at failure was obtained from the load–deflection curves, and Young’s modulus was determined based on the slope of the initial linear elastic portion of the load–deflection curve. Each group consisted of at least twenty samples.

The tensile strength tests were conducted using flat, dumbbell-shaped specimens according to ASTM D638 standard. These specimens had a gauge length of 32 mm, with the central area being 8 mm long, 3 mm wide, and 3 mm thick, which were tested using a Shimadzu AGS-10kNX. The tensile stress (σ) and tensile strain (ε) were calculated using the following formulas: σ = P/wh, ε = ∆L/L, where P represents the peak load and w and h are the width (mm) and the thickness (mm) of the specimen. L and ∆L represent the gage length and the tensile axial elongation, respectively. Finally, the elastic modulus (E) was obtained from the equation E = σ/ε. Twenty samples were used per group.

A rectangular bar specimen measuring 5 × 2 × 40 mm^3^ was used for three-point bending testing following ASTM D790 standard. The test was conducted using a Shimadzu AGS-10kNX with the load applied over a 32 mm span. The strength value of each specimen was calculated using the equation σ = 3 PL/2 wh^2^, where P is the peak load (N), L is the distance (mm) between the supports, w is the width (mm), and h is the height (mm) of the specimen. Young’s bending modulus (E) was calculated using the equation E = mL^3^/4 wh^3^, where m is the slope of the initial linear elastic portion of the load–deflection curve. The reported data for each composition represented the mean of twenty independent measurements.

### 2.5. Cytotoxicity

L929 fibroblasts (BCRC RM60091, Hsinchu, Taiwan) were used to assess the cytotoxicity of samples treated at 240 °C, according to ISO 10993-5 standard. Before cell incubation, samples (9 mm diameter, 1 mm thickness) were sterilized by washing with 75% ethanol and then exposed to UV light overnight. Dulbecco’s modified Eagle medium (DMEM; Gibco, Langley, OK, USA) containing 10% fetal bovine serum (FBS; Gibco) and 1% penicillin/streptomycin solution (Gibco) without sample was used as the negative control. A culture medium containing 10% dimethyl sulfoxide (DMSO; Sigma-Aldrich) was used as the positive control. After 12, 24, and 48 h of L929 culture using 5000 cells per well in a 48-well plate, the cytotoxicity was evaluated using the MTT (3-(4,5-dimethylthiazol-2-yl)-2,5-diphenyltetrazolium bromide; Sigma-Aldrich) assay. Cell viability was standardized to the negative control based on absorbance measured using a BioTek Epoch spectrophotometer (Winooski, VT, USA) at 570 nm. Results were from three separate experiments.

### 2.6. Bacteria Response

The effects of PSF-based samples on bacterial responses were studied using *E. coli* (ATCC 8739, Hsinchu, Taiwan) and *S. aureus* (ATCC 25923, Hsinchu, Taiwan) after sterilization. Disk samples measuring 9 mm in diameter and 1 mm in thickness were placed in 48-well culture plates, and 1 mL of bacteria were seeded at a density of 10^5^ CFU/mL in Bacto tryptic soy broth (Beckton Dickinson, Sparks, MD, USA) for a culture period of 3, 6, 24, and 48 h. The culture broth in a plate without composite samples was used as a control. Subsequently, the Alamar Blue (Invitrogen, Grand Island, NY, USA) assay was used to assess bacterial growth on the samples. At the end of the culture period, the samples were washed with phosphate buffer solution (PBS, pH 7.4) twice to remove loosely adherent bacteria from the sample surfaces. Alamar Blue solution was added to each well for a 30-min reaction and then examined using a BioTek Epoch spectrophotometer at 570 nm. The bacteriostatic ratio (%) was calculated as follows: (absorbance on the control–absorbance on the composite sample)/absorbance on the control × 100% [31]. The results were obtained in triplicate.

### 2.7. Statistical Analysis

The utilization of one-way analysis of variance (ANOVA) allowed for the comprehensive evaluation of differences among means in the measured data. Subsequently, the application of Duncan’s multiple comparison testing efficiently determined the significance of standard deviations within the measured data from each specimen across diverse experimental conditions. In all instances, results were deemed statistically significant when the *p*-value was less than 0.05.

## 3. Results

### 3.1. Morphology of Raw Materials

To better understand the characteristics of composites, it is essential to comprehend the structure of the raw materials first. Figure 1 displays the morphology of different materials, including recycled PSF fiber, PSF-derived nanoparticles, and CaSi particles. The optical image of PSF from a recycled dialysis tube revealed long fiber shapes (Figure 1A). After fracturing, the SEM micrographs showed short fibers with a hollow structure (Figure 1B), and the fiber surface contained many 1 μm-diameter holes (Figure 1C). The PSF-precipitated particles, whether from recycled (Figure 1D) or commercial raw materials (Figure 1E), showed an agglomeration of about 50 nm in size. Additionally, the CaSi filler shows an aggregate of 200 nm particles, as shown in Figure 1F.

### 3.2. Phase Composition

**Figure 2:**
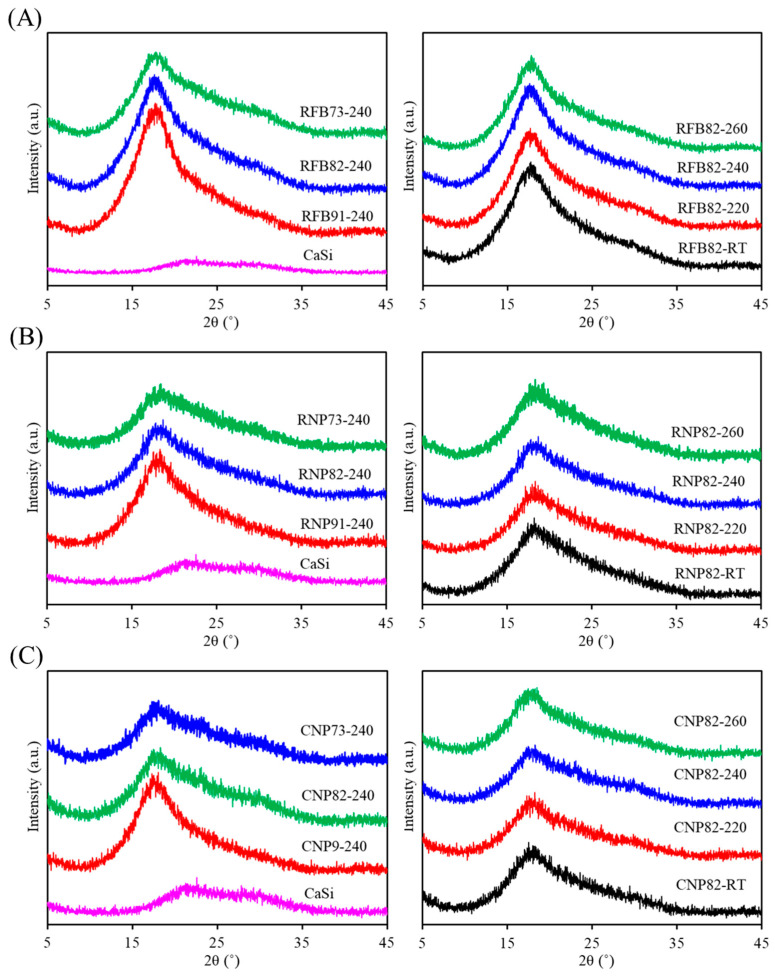
XRD patterns of (**A**) RFB-based, (**B**) RNP-based, and (**C**) CNP-based composites with different matrix/ceramic ratios after heat treatment at different temperatures. (RT: room temperature without heat treatment).

Figure 2 illustrates the phase revolution of composites, including CaSi filler, three different PSF structures (fiber and particle) or sources (medical waste and commercial product) mixed with varying ratios of CaSi fillers after being treated at firing temperatures. In Figure 2A, the CaSi powder exhibited broad and diffuse peaks at approximately 2θ = 20–30°, consisting of amorphous SiO_2_, CaSiO_3_, and CaCO_3_ phases [35]. The recycled fiber (RFB)-based composites display a broad diffraction peak around 17.6°, unique to amorphous materials and attributed to the PSF phase [41,42]. As the amount of CaSi increased, the peak intensity of PSF decreased. The firing temperature appeared to have no impact on the phase change, resulting in the same diffraction peak and intensity. After compounding CaSi particles, RNP-based (Figure 2B) and CNP-based composites (Figure 2C) displayed a similar trend in phase composition to the RFB-based composites. Specifically, there was an increase in the broad peak observed at 2θ = 20–30° as the CaSi content increased.

**Figure 3:**
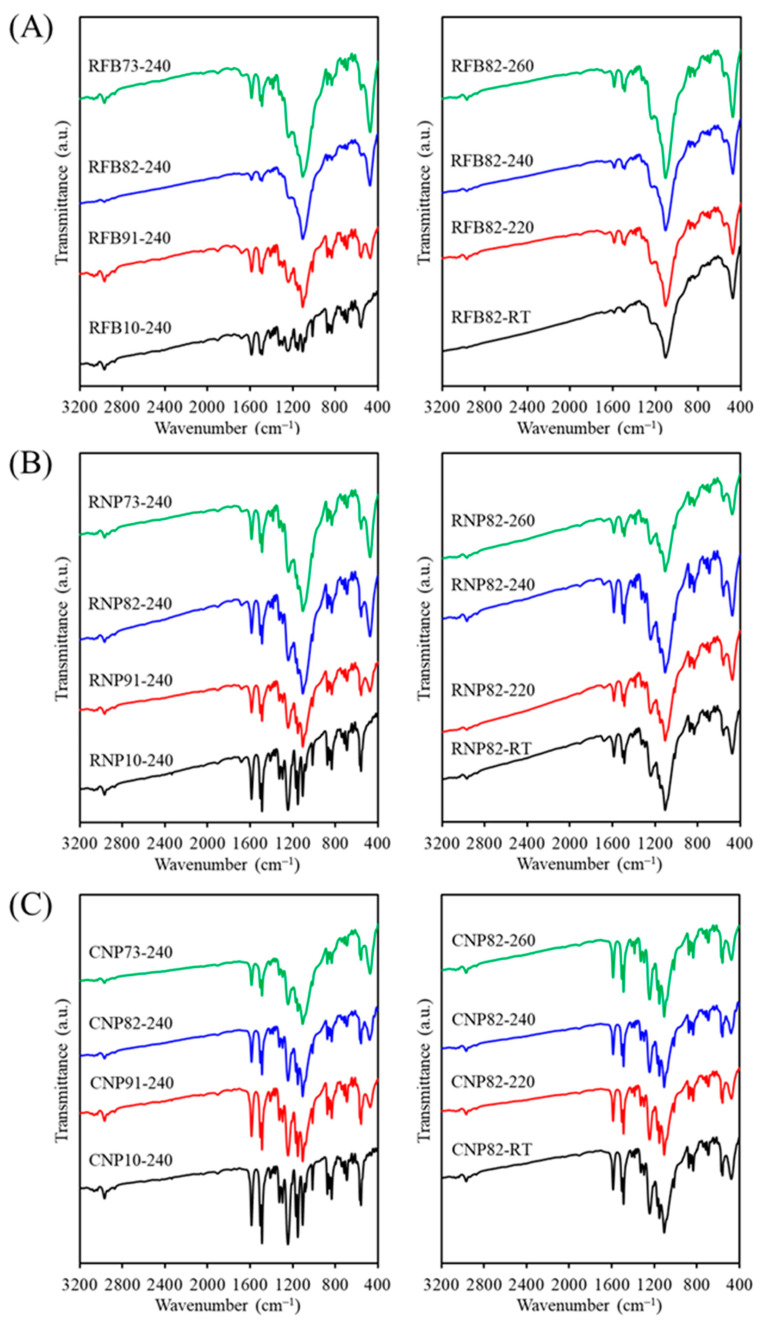
FTIR spectra of (**A**) RFB-based, (**B**) RNP-based, and (**C**) CNP-based composites with different matrix/ceramic ratios after heat treatment at different temperatures. (RT: room temperature without heat treatment).

The FTIR spectra of the RFB groups with and without CaSi contents showed variations after being fired at various temperatures (Figure 3A). The 240 °C-treated recycled PSF samples without CaSi (RFB-240) exhibited characteristic peaks and bands of PSF groups. The aromatic C–H stretching vibrations marked its presence by an absorption band at 3100–2940 cm^−1^ [22,41,43]. The bands at 1587–1488 cm^−1^ were attributed to C_6_H_6_ ring stretching [40], and those at 1385–1364 cm^−1^ were due to gem-dimethyl groups of the PSF matrix [41]. The bands at 1326 cm^−1^ and 1295 cm^−1^ corresponded to asymmetric stretching of C–SO_2_–C [22,43,44], whereas the band at 1151 cm^−1^ came from symmetric stretching of the sulfone groups in the C–SO_2_–C bridge [43,44,45]. The asymmetric stretching of the C–O–C group occurred at 1246 cm^−1^ and 1014 cm^−1^ [22,41,44,45], and the bands at 1100–1000 cm^−1^ were attributed to the symmetrical stretching vibration of the sulfoxide (S=O) group [22,41]. The 874, 854, and 834 cm^−1^ peaks were assigned to the aromatic C–H bending [44]. The 740–690 cm^−1^ peaks were attributed to the C–S stretching vibrational modes [22], and the peak at 558 cm^−1^ was ascribed to the C–C vibrational mode.

When CaSi fillers were added to the PSF matrix, subtle differences can be observed between the samples. The CaSi material displayed a characteristic SiO_4_ asymmetric stretching and the Si–O–Ca vibrational mode over a wide wavenumber range of 1250 to 900 cm^–1^ [35]. However, these bands overlapped with some characteristic bands of the PSF matrix. It is important to mention that the sharp band at 473 cm^–1^ was ascribed to a rocking mode of Si–O–Si of the CaSi component [35]. With increased CaSi content in the composite, the peak intensity at 473 cm^−1^ increased, while the 558 cm^−1^ peak from the PSF matrix decreased. Similar to the XRD results, variations in firing temperature did not lead to noticeable changes in the FTIR spectra. In comparing RFB and RNP samples, the band intensity of the RNP10 sample without CaSi fillers was higher than that of RFB10 at the same firing temperature of 240 °C (Figure 3B), but the band positions did not change. As depicted in Figure 3C, the FTIR spectra of all CNP samples show no differences from those of RNP groups, irrespective of the composition effect and temperature parameters.

### 3.3. Formability of Composites

**Figure 4:**
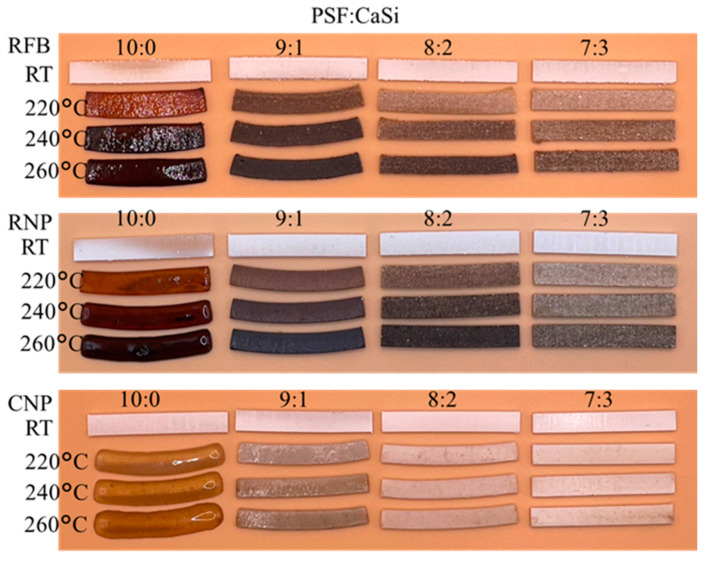
Images photographed for the dimension changes in RFB, RNP, and CNP samples after different firing temperatures. RT: room temperature without heat treatment.

In Figure 4, the formability of various PSF/CaSi composites is shown to represent the structural stability. The accompanying images vividly illustrate the temperature effect of different composite formulations. Upon treatment at 220–260 °C, it is evident that the pure PSF samples from three sources underwent shape transformation and shrinkage. Notably, with an increase in the content of CaSi within the PSF matrix, the PSF/CaSi composites exhibited remarkable dimensional and shape retention, particularly in the case of samples with an 8:2 and 7:3 ratio of PSF to CaSi.

### 3.4. Morphology of Composites

**Figure 5:**
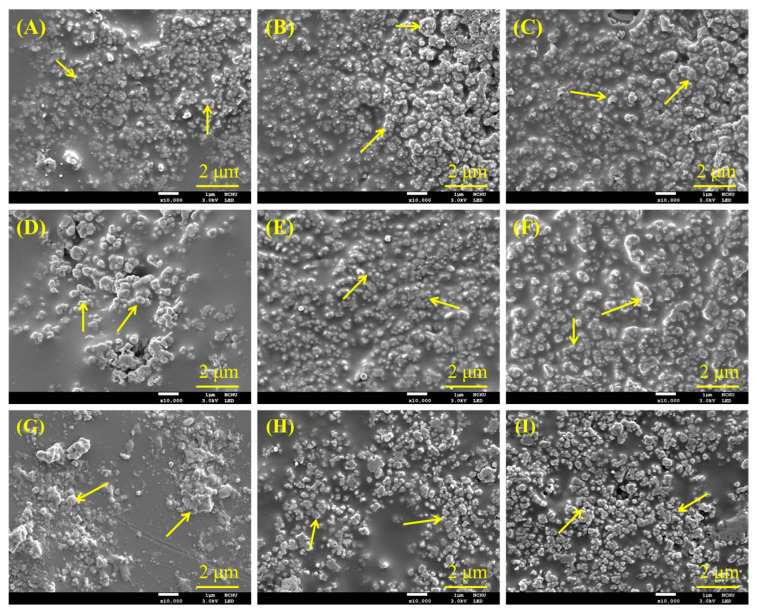
SEM micrographs of (**A**) RFB91, (**B**) RFB82, (**C**) RFB73, (**D**) RNP91, (**E**) RNP82, (**F**) RNP73, (**G**) CNP91, (**H**) CNP82, and (**I**) CNP73 composites after heat treatment at 240 °C. Arrows indicate CaSi particles.

SEM was further used to observe the morphology of various PSF composites. Figure 5 displays the surface SEM micrographs of composite materials with different PSF sources or structures, each loaded with three different amounts of CaSi after heat treatment at 240 °C. The granular particles marked by arrows were the CaSi component incorporated into the smooth structure. This smooth structure resulted from the softening of the PSF during heat treatment, independent of the PSF sources or structure. As the CaSi content increased, the smooth structure of the PSF matrix decreased.

### 3.5. Mechanical Properties

#### 3.5.1. Compressive Properties

**Figure 6:**
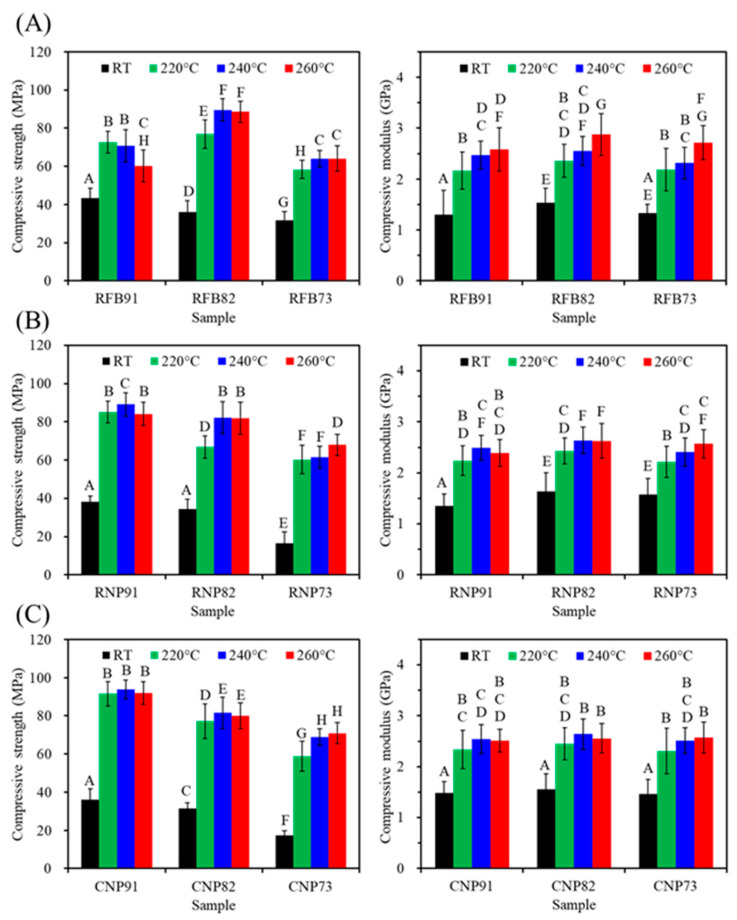
Compressive strength (left) and modulus (right) of (**A**) RFB-, (**B**) RNP-, and (**C**) CNP-based composites before (RT) and after different heat treatments (220, 240, and 260 °C). Statistical comparisons were conducted among all samples, and different capital letters indicated significant differences at *p* < 0.05.

Figure 6 shows the compressive strength and modulus changes in three different PSF-based composites containing various amounts of CaSi fillers before and after exposure to different heat temperatures. Clearly, compared to the untreated groups (RT), the elevated heat treatment temperature would increase the strength value to some degree. As expected, the PSF/CaSi composite displayed a remarkable increase in strength due to the increased binding of the PSF matrices after annealing. Treatment temperature, PSF structure, and PSF/CaSi mixing ratios affected the compressive strength, eliciting non-monotonous changes. In the case of RFB groups, the composite RFB82 containing 20 vol% CaSi exhibited a significantly (*p* < 0.05) higher compressive strength value of 77–90 MPa compared to the 10 vol% (RFB91) with 60–73 MPa and 30 vol% CaSi (RFB73) composites with 58–64 MPa (Figure 6A). Composites from PSF nanoparticles (RNP and CNP groups) also exhibited reduced strength values due to elevated CaSi content. For example, at a treatment temperature of 220 °C, RNP91 demonstrated a compressive strength of 85 MPa, surpassing RNP73 with a strength of 60 MPa (Figure 6B). Likewise, as the CaSi content increased from 10 vol% to 30 vol%, the compressive strength of CNP composites decreased from 92 MPa to 59 MPa (Figure 6C). It is essential to consider the elasticity of an implant material when tailoring its mechanical properties. Regarding the compressive modulus, all heat-treated composites were about 2.5 GPa, showing minimal variations after different treatment temperatures and composition adjustments.

#### 3.5.2. Tensile Properties

**Figure 7:**
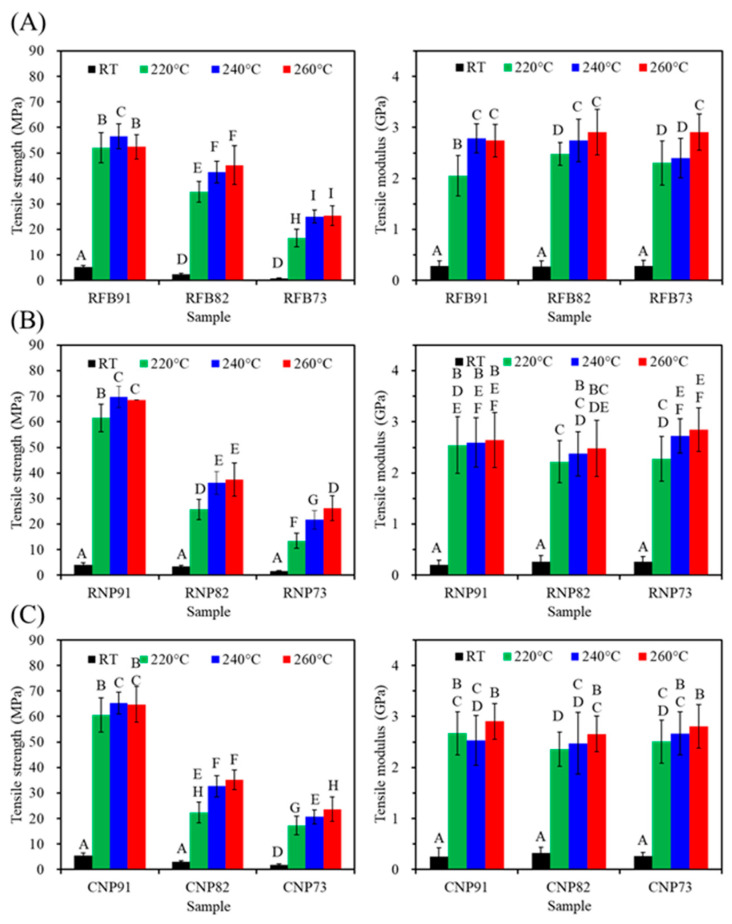
Tensile strength (left) and modulus (right) of (**A**) RFB-, (**B**) RNP-, and (**C**) CNP-based composites before (RT) and after different heat treatments (220, 240, and 260 °C). Statistical comparisons were conducted among all samples, and different capital letters indicated significant differences at *p* < 0.05.

According to Figure 7, PSF-based composites from three sources showed a significant decrease (*p* < 0.05) in tensile strength with increased CaSi content. It is evident that the firing temperature of 220–260 °C has a substantial impact on strength, especially when compared to the untreated material. Furthermore, except for the 91 groups (RFB91, RNP91, and CNP91), higher firing temperatures increased the tensile strength of the composites in the 82 and 73 groups. In the case of RFB groups (Figure 7A), the tensile strength values of RFB82 were 35, 42, and 45 MPa after 220, 240, and 260 °C treatments, indicating a significant increase (*p* < 0.05). Similar trends were observed for RNP (Figure 7B) and CNP groups (Figure 7C). On the other hand, the modulus remained at about 3 GPa, almost independent of the composite’s composition and firing temperature.

#### 3.5.3. Three-Point Bending Properties

**Figure 8:**
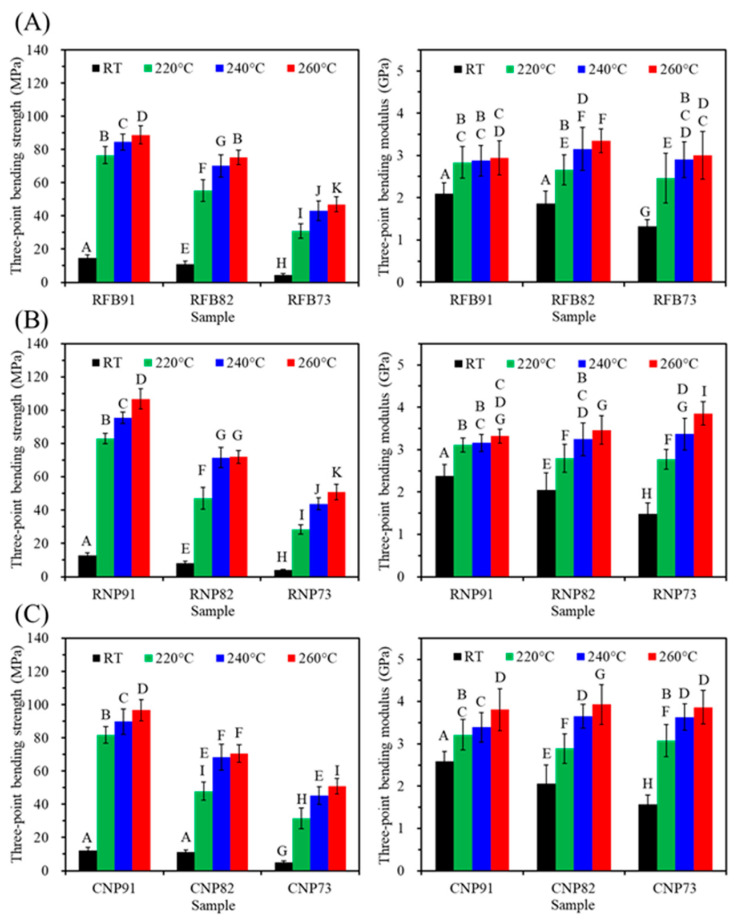
Three-point bending strength (left) and modulus (right) of (**A**) RFB-, (**B**) RNP-, and (**C**) CNP-based composites before (RT) and after different heat treatments (220, 240, and 260 °C). Statistical comparisons were conducted among all samples, and different capital letters indicated significant differences at *p* < 0.05.

The three-point bending test is recommended for assessing both tensile and compressive stress on a sample, making it suitable for comparing the mechanical properties of polymer-based materials. Figure 8 shows the changes in the three-point bending strength of various PSF-based composites when they underwent heat treatment at different temperatures. The composites exhibited higher bending strength at higher firing temperatures. However, a higher content of CaSi led to a lower tensile strength value of the PSF-based composites. For instance, at a heat treatment temperature of 240 °C, the bending strength of RFB91, RFB82, and RFB73 was 84, 70, and 43 MPa (Figure 8A), respectively. In the case of the RNP group (Figure 8B), adding CaSi up to 20 and 30 vol% resulted in bending strength values of 72 and 44 MPa, respectively. Similarly, the CNP group exhibited the corresponding 68 and 45 MPa (Figure 8C). Notably, the bending modulus of all composites was in the range of 2.5–3.8 GPa, and the firing temperature positively impacted the modulus.

### 3.6. L929 Cytotoxicity

**Figure 9:**
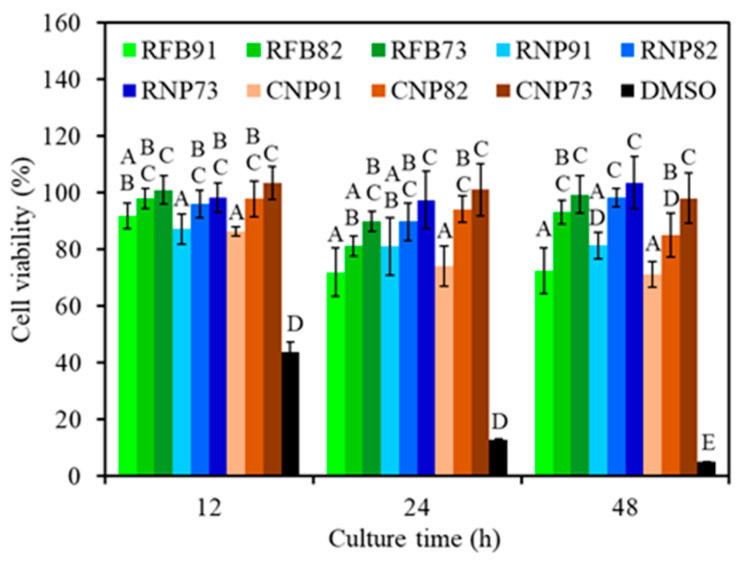
Cytotoxicity of various composites treated at 240 °C and seeded with L929 cells at different time points. Statistical comparisons were conducted between samples incubated for the same duration, and different capital letters were used to denote significant differences at *p* < 0.05. Except for the DMSO positive control, all composite samples exhibited more than 70% cell viability.

The L929 cytotoxicity of all samples is depicted in Figure 9. Not surprisingly, the positive control (DMSO) induced significant cytotoxicity with the increasing culture time. On the contrary, all composite samples exhibited more than 70% cell viability during 12–48 h of culture. The viability of L929 cells indicated that all 91 groups (RFB91, RNP91, and CNP91) had significantly lower (*p* < 0.05) viability compared to 73 groups (RFB73, RNP73, and CNP73). It is worth highlighting that 82 groups showed comparable cell viability to 73 groups, signifying no significant difference (*p* > 0.05) between the two groups.

### 3.7. Bacterial Response

**Figure 10:**
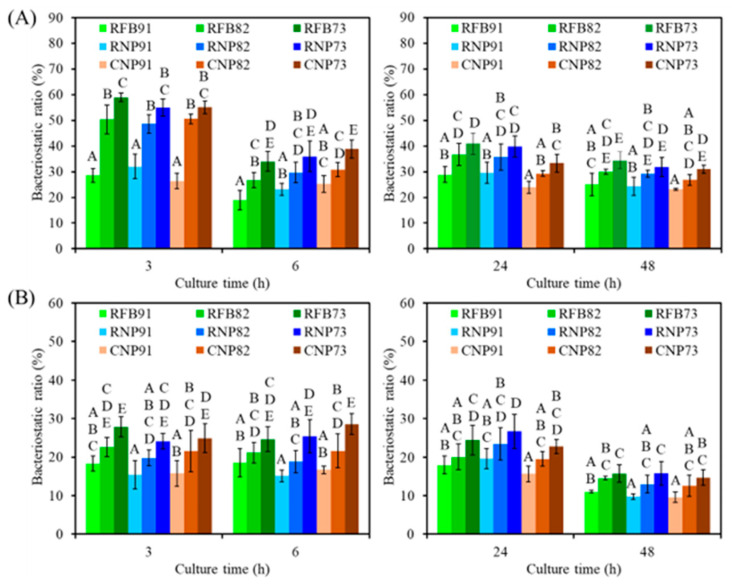
The bacteriostatic ratio of the various samples against (**A**) *E. coli* and (**B**) *S. aureus* bacterial species after culture for short-term (3 h and 6 h) and long-term time points (24 h and 48 h). Statistical comparisons were made between samples incubated at the same time point, and different capital letters indicated significant differences at *p* < 0.05.

To verify the antibacterial activity of CaSi-containing PSF composites, the bacteriostatic ratio (%) against *E. coli* and *S. aureus* was assessed. When *E. coli* was cultured on the surface of different composites for 3 h (Figure 10A), all composites displayed amount-dependent bacteriostatic ratios. For example, RFB91, RFB82, and RFB73 had bacteriostatic ratios of 29%, 50%, and 59%, respectively. Similarly, RNP73 and CNP73 showed a significant 57% and 55% increment (*p* < 0.05) in the bacteriostatic ratio, respectively, compared to the corresponding RNP91 and CNP91 after 6 h. At hours 24 and 48, the composites with 73 ratios (RFB73, RNP73, and CNP73) demonstrated a significant (*p* < 0.05) increase in the bacteriostatic ratio compared to composites with 91 ratios (RFB91, RNP91, and CNP91). In the case of *S. aureus* (Figure 10B), higher CaSi content in the composites led to significantly increased bacteriostatic ratios during the seeding periods. However, the sources of PSF did not impact the antibacterial efficacy. In contrast to the 91 groups, there was almost no significant (*p* < 0.05) difference between the 82 and 73 groups.

## 4. Discussion

Reusing recycled PSF waste materials is attractive from ecological and economic perspectives [46,47,48]. In this study, long fibers of PSF from recycled dialysis tubes were cut into short fibers and can be further processed into nanoparticles, mirroring the properties of commercial raw materials. Although there were two types of PSF (RNP and CNP) nanoparticles, from the recycling and green chemistry perspective, the preference is to reuse RFB. Therefore, the CaSi particles were not further milled to below 100 nm nanoparticles. Our study delved into the composition and microstructure of PSF-based composites upon including CaSi in the PSF matrix. Since the CaSi filler was sintered at 800 °C before being incorporated into the PSF matrix, it is reasonable to expect that the low heat treatment temperature of 220–260 °C will not affect the phase combination. Additionally, adding CaSi did not lead to new phase formation or wavenumber shifts, as evidenced by XRD and FTIR analyses, signifying no chemical interaction between PSF chains and CaSi functional groups. The structural observations unveiled that the PSF matrix and CaSi filler were mechanically interlocked due to the liquefaction of PSF fibers or nanoparticles during heat treatment. Notably, the inclusion of CaSi significantly enhanced the dimensional stability of the composite during the firing treatment, underscoring its ability to bolster the thermal stability of the composite. Another noteworthy advantage of recycled PSF long fibers was their facilitation of PSF nanoparticle preparation due to their hollow structure, enabling more straightforward dissolution than commercial products with denser structures.

The strength of an implant is crucial for load-bearing applications. Adequate mechanical properties in cortical bone grafts and fracture fixation devices are essential for promoting bone regeneration, supporting the strength of the healing tissue, and preventing displacement [15]. To this end, this study examined the mechanical properties of PSF-based composites, which are crucial for various clinical applications such as spacers, artificial vertebrae, intervertebral discs, iliac crests, dental implants, and bone fracture fixation [12,49,50]. Hence, three different stress modes (Table 1) were utilized to assess these composites, with their mechanical properties influenced by the chemical composition, microstructure, and heat treatment [12,51]. For example, Jeyachandran et al. found that adding 20 wt% CaSi-based bioactive glass increased HDPE’s compressive strength to 26 MPa and modulus to 0.6 GPa [52]. The molecular structure of the amorphous PSF matrix can be enhanced through annealing to improve mechanical strength. Regarding compressive strength, the chemical precipitated CaSi particles used as noncovalent connectors between PSF were found to be fragile despite ceramics inherently possessing high compressive strength. This fragility may explain why the compressive strength of the PSF-based composites did not increase with higher CaSi content. Higher firing temperature may increase strength, particularly for composites with higher CaSi content, potentially due to the liquid phase sintering of PSF. Regardless of the PSF structure and firing temperatures, the composites with 20% CaSi exhibited a compressive strength of approximately 80 MPa, as listed in Table 1. Overall, the 240 °C-treated composites consistently demonstrated good compressive strength across all samples.

The tensile properties of a material are vital for its clinical application. It is fascinating that the tensile strength of PSF-based composites remained unaffected by the PSF structure, whether from recycled fiber or commercial products. Just like the research of Yu et al., recycled PSF from wasted nonwovens exhibited tensile properties similar to virgin PSF plastics, emphasizing the recycling value of PSF thermoplastics [46]. The current study found that higher CaSi content reduced the tensile or bending strength of the composites, as reported in other studies [27,53]. Cao et al. reported that the remarkable tensile strength and bending strength of pure PEEK stood at 93 MPa and 141 MPa, respectively. However, when 30 wt% of CaSiO_3_ was introduced to PEEK, the resulting composite experienced a significant decline in both tensile and bending strength, with 41% and 45% reduction rates, respectively [27]. This is due to the low tensile resistance of CaSi particles being pulled away from the PSF matrix. Furthermore, the decline in the strength of polymer-based composites could be attributed to partial cracks at the interface between the polymer matrix and the ceramic filler caused by the tension during measurement [54]. Additionally, the current XRD and SEM results indicated that poor crystallinity and agglomerations of CaSi fillers might be important factors affecting the mechanical properties. To enhance the mechanical properties of polymeric-based composites, it is crucial to reduce agglomerations, improve adhesion, and enhance the crystallinity of ceramic fillers [52,55,56]. The uniform distribution of bioactive glass within the HDPE can enhance the bending and compressive strength of the composites [52]. Costa et al. used high-energy ball milling to achieve a homogeneous microstructure with good interfacial adhesion between PEEK and HA, increasing compressive strength [55]. Nevertheless, with the addition of 20% CaSi into the RFB matrix and treatment at 240 °C, its tensile strength of 42 MPa was higher than that of the composite containing 10 wt% HA, 10 wt% ZnO, and 80 wt% HDPE (36 MPa) [26], as well as UHMWPE containing carbonated HA and collagen (31 MPa) [57]. On the other hand, the three-point bending strength of PSF-based composites containing 20% CaSi from three types of PSF heat-treated at 240 °C exceeded 60 MPa, falling within the reported bending strength range of cortical bone (50–150 MPa) [58]. This suggests that the developed PSF/CaSi hybrid composites with such mechanical properties could potentially be used in load-bearing applications. However, further investigations are essential to explore the use of binding agents such as silanes, which have shown promise in enhancing the mechanical properties of composites, to strengthen the mechanical properties of PSF-based composites.

The elasticity of materials is a crucial aspect of their mechanical properties. Pure PSF has an elastic modulus of 2.2–2.7 GPa [59,60], which is lower than that of cortical bone (7–30 GPa) [12]. Generally, ceramics have a higher elastic modulus than polymers. By adding CaSi particles to the three PSF types, we can increase the elastic modulus of the composites. Indeed, the current PSF-based composites had a modulus of approximately 3 GPa under different stress modes. Oréfice et al. also noted that increasing bioactive glass in PSF-based composites could lead to lower flexural strength but an improved modulus [61]. Adding 30 wt% CaSiO_3_ particles to PEEK increased the tensile and bending modulus to 4.9 GPa and 5.6 GPa, about 67% and 44% higher than PEEK [27]. Similarly, the PEEK containing 5 wt% CaSi-based bioactive glass samples, printed at laser powers of 23 W, improved the tensile modulus to 3.7 GPa from the original 3.5 GPa [53]. Selecting a bioactive material with an elastic modulus similar to the bone being replaced or fixed is paramount. This ensures a uniform stress distribution across the bone and implant interface, which is crucial in avoiding implant loosening during long-term service and reducing stress shielding [62,63]. Incorporating tougher bioceramics in the PSF-based composites may remarkably enhance the elastic modulus. Conversely, research by Goodship et al. indicated that using low-modulus devices could facilitate the formation of external callus tissue, decrease surface stress, and transfer maximum stress from the surface to the interior of a bone implant, potentially leading to faster healing [64]. Moreover, flexible polymer-based implants offer adequate stability for the bone union during the initial stage of fracture healing and allow for some degree of mechanical stimulation at the fracture site [11,65]. These findings and the potential of the current PSF-based composites provide hope for a beneficial role in fracture healing. Based on all the shape and strength results, a firing temperature of 240 °C was selected for further biological tests.

Ensuring the biocompatibility of the material is a critical initial step in understanding its biological performance, such as safety and effectiveness. In vitro cytotoxicity testing is often used to assess this before proceeding to further osteogenic evaluation and animal studies. PSF has been proven to support the growth of various human and animal cells, including periodontal ligament fibroblasts [66], rat hepatocytes [67], liver stem cells [68], and human osteoblast-like MG63 cells [69]. Nevertheless, it is imperative to verify the biocompatibility of PSF-based composites. The current cytotoxicity results for L929 indicate that all composites were non-cytotoxic as their viability exceeded 70% (Table 1), meeting the standard ISO 10993-5 definition. More importantly, CaSi promoted the cell viability of the PSF-based composites in a dose-dependent manner, as reported in a previous study [51]. The literature indicates that CaSiO_3_ can promote the growth of MC3T3-E1 cells when cultured on the surface of the PEEK/CaSiO_3_ composite [27]. It is also noteworthy that the different types of PSF did not affect cytotoxicity, reinforcing the reliability of our findings. Nonetheless, further investigation into the osteogenic properties of these composites is crucial despite the confirmed biocompatibility.

The leading causes of joint arthroplasty revision are infection and mechanical loosening [70,71]. Most early periprosthetic joint infections are believed to happen during implantation and are caused by skin bacteria or external sources from the operating room [71]. Biomaterial-associated infections often require prolonged treatment and additional surgical procedures and can lead to poor functional outcomes [72]. This underscores the urgent need for new materials to combat these infections. One approach to prevent bacterial adhesion to bone implants is to develop non-antibiotic antibacterial materials to avoid drug resistance. Since PSF lacks antibacterial activity [42,73], it is essential to incorporate antibacterial additives to address this deficiency [41,74,75]. As expected, the presence of CaSi in the PSF-based composites reduced the number of viable bacteria to some degree, likely due to their alkalinity. CaSi releases hydroxide ions (OH^–^) when exposed to a liquid environment, effectively damaging bacterial cytoplasmic membranes, structural proteins, and DNA [31]. Antibacterial mechanisms of CaSi may be due to the production of reactive oxygen species (ROS) in addition to alkaline pH, as mentioned in a previous report [35]. ROS, including superoxide anions, hydroxyl radicals, singlet oxygen, and hydrogen peroxide, are by-products of cell oxidative metabolism. A higher CaSi content is believed to contribute to enhanced antibacterial activity in PSF-based composites. Furthermore, the antibacterial effect of the CaSi-containing composites remained consistent across different bacterial strains, PSF types, and seeding time intervals. Enhancing antibacterial properties was a key goal in designing these load-bearing materials. However, while including CaSi has improved PSF’s antibacterial capabilities, it alone was not enough to completely inhibit/kill bacterial growth. Therefore, additional strategies, such as implementing an antibacterial coating layer, should be considered to achieve optimal antibacterial properties.

## 5. Conclusions

This study highlighted the upcycling potential of PSF medical waste as a matrix filled with CaSi bioceramics. This discovery could bring hope to the field of load-bearing composites. Despite a decrease in mechanical strength with increased CaSi content, there was a simultaneous improvement in the biocompatibility and antibacterial activity of the PSF-based composites. The 20% CaSi-containing PSF-based composites treated at 240 °C have emerged as a promising material for cortical bone defect and bone fracture fixation applications due to their mechanical properties and antibacterial activity against *E. coli* and *S. aureus* bacterial species. Ongoing research on osteogenesis and long-term in vitro studies will further confirm the numerous benefits of these recycled PSF waste plastics, which could serve as a foundational material, offering hope for developing new and improved medical materials.

## Figures and Tables

**Table 1:** Mechanical properties and L929 viability of various PSF-based composites treated at 240 °C.

Sample Code	Compressive		Tensile		Bending		Cell Viability (%)		
	Strength (MPa)	Modulus (GPa)	Strength (MPa)	Modulus (GPa)	Strength (MPa)	Modulus (GPa)	12 h	24 h	48 h
RFB									
RFB91	71 ± 8	2.5 ± 0.3	57 ± 5	2.8 ± 0.3	84 ± 5	2.9 ± 0.4	92 ± 5	72 ± 8	73 ± 8
RFB82	90 ± 6	2.6 ± 0.3	42 ± 4	2.8 ± 0.4	70 ± 7	3.2 ± 0.5	98 ± 4	81 ± 4	93 ± 4
RFB73	64 ± 6	2.3 ± 0.3	25 ± 3	2.4 ± 0.4	43 ± 6	2.9 ± 0.4	101 ± 5	90 ± 4	99 ± 7
RNP									
RNP91	89 ± 7	2.5 ± 0.3	70 ± 4	2.6 ± 0.5	96 ± 4	3.2 ± 0.2	87 ± 5	81 ± 10	81 ± 4
RNP82	82 ± 6	2.6 ± 0.3	36 ± 4	2.4 ± 0.4	72 ± 6	3.3 ± 0.4	96 ± 5	90 ± 7	98 ± 3
RNP73	62 ± 6	2.4 ± 0.3	22 ± 4	2.7 ± 0.3	44 ± 4	3.4 ± 0.4	98 ± 5	98 ± 10	104 ± 9
CNP									
CNP91	93 ± 5	2.5 ± 0.3	65 ± 4	2.5 ± 0.5	90 ± 8	3.4 ± 0.4	86 ± 2	74 ± 7	71 ± 5
CNP82	82 ± 8	2.6 ± 0.3	33 ± 4	2.5 ± 0.6	68 ± 8	3.7 ± 0.3	98 ± 6	94 ± 5	85 ± 8
CNP73	69 ± 4	2.5 ± 0.3	21 ± 3	2.7 ± 0.4	45 ± 5	3.6 ± 0.3	103 ± 6	101 ± 9	98 ± 9

## Data Availability

The original contributions presented in the study are included in the article; further inquiries can be directed to the corresponding author.

## References

[1] Jambeck J., Geyer R., Wilcox C., Siegler T.R., Perryman M., Andrady A., Narayan R., Law K.L. (2015). Plastic Waste Inputs from Land into the Ocean. Science.

[2] Liu X., Li Y., Fang X., Zhang Z., Li S., Sun J. (2022). Healable and recyclable polymeric materials with high mechanical robustness. ACS Mater. Lett..

[3] https://www.tsn.org.tw/twrds.html?page=&year=2022#.

[4] Nakashima A., Ogata S., Doi S., Yamahira M., Naraki S., Takasugi N., Ohmoto T., Ito T., Masaki T., Yorioka N. (2006). Performance of polysulfone membrane dialyzers and dialysate flow pattern. Clin. Exp. Nephrol..

[5] Azizah D.A., Kusworo T.D., Kumoro A.C. (2024). Developing UV-light driven photocatalytic PSf/Ni-doped ZnO/PDA membrane with superior antifouling, self-cleaning, and self-protecting performances for handmade batik wastewater treatment. Mater. Today Sustain..

[6] Filimon A., Albu R.M., Stoica I., Avram E. (2016). Blends based on ionic polysulfones with improved conformational and microstructural characteristics: Perspectives for biomedical applications. Compos. B.

[7] Stannat S., Bahlmann J., Kiessling D., Koch K., Deicher H., Peter H.H. (1985). Complement activation during hemodialysis. Comparison of polysulfone and cuprophan membranes. Contrib. Nephrol..

[8] Schaefer R.M., Heidland A., Hörl W.H. (1985). Release of leukocyte elestase during hemodialysis. Effect of different dialysis membranes. Contrib. Nephrol..

[9] Nechifor G., Totu E.E., Nechifor A.C., Isildak I., Oprea O., Cristache C.M. (2019). Non-resorbable nanocomposite membranes for guided bone regeneration based on polysulfone-quartz fiber grafted with nano-TiO_2_. Nanomaterials.

[10] Jacobs J.J., Gilbert J.L., Urban R.M. (1998). Corrosion of metal orthopaedic implants. J. Bone Jt. Surg. Am..

[11] Kettunen J., Makela E.A., Miettinen H., Nevalainen T., Heikkilä M., Pohjonen T., Törmälä P., Rokkanen P. (1998). Mechanical properties and strength retention of carbon fibre-reinforced liquid crystalline polymer (LCP/CF) composite: An experimental study on rabbits. Biomaterials.

[12] Wei C.K., Ding S.J. (2016). Acid-resistant calcium silicate-based composite implants with high-strength as load-bearing bone graft substitutes and fracture fixation devices. J. Mech. Behav. Biomed. Mater..

[13] Chen C.-F., Chou Y.-S., Lee T.-M., Fu Y.-C., Ou S.-F., Chen S.-H., Lee T.-C., Wang Y.-H. (2024). The uniform distribution of hydroxyapatite in a polyurethane foam-based scaffold (PU/HAp) to enhance bone repair in a calvarial defect model. Int. J. Mol. Sci..

[14] Bergsma E.J., Rozema F.R., Bos R.M., De Bruijn W.C. (1993). Foreign body reactions to resorbable poly(l-lactide) bone plates and screws used for the fixation of unstable zygomatic fractures. J. Oral Maxillofac. Surg..

[15] Ahmed I., Jones I.A., Parsons A.J., Bernard J., Farmer J., Scotchford C.A., Walker G.S., Rudd C.D. (2011). Composites for bone repair: Phosphate glass fibre reinforced PLA with varying fibre architecture. J. Mater. Sci. Mater. Med..

[16] Navarro M., Michiardi A., Castaño O., Planell J.A. (2008). Biomaterials in orthopaedics. J. R. Soc. Interface.

[17] Mano J.F., Sousa R.A., Boesel L.F., Neves N.M., Reis R.L. (2004). Bioinert, biodegradable and injectable polymeric matrix composites for hard tissue replacement: State of the art and recent developments. Comp. Sci. Tech..

[18] Samantaray P.K., Little A., Haddleton D.M., McNally T., Tan B., Sun Z., Huang W., Ji Y., Wan C. (2020). Poly(glycolic acid) (PGA): A versatile building block expanding high performance and sustainable bioplastic applications. Green Chem..

[19] Wang M., Bonfield W. (2001). Chemically coupled hydroxyapatite–polyethylene composites: Structure and properties. Biomaterials.

[20] Dalby M.J., Kayser M.V., Bonfield W., Di Silvio L. (2002). Initial attachment of osteoblasts to an optimised HAPEX topography. Biomaterials.

[21] Huang H., Liu X., Wang J., Suo M., Zhang J., Sun T., Wang H., Liu C., Li Z. (2024). Strategies to improve the performance of polyetheretherketone (PEEK) as orthopedic implants: From surface modification to addition of bioactive materials. J. Mater. Chem. B.

[22] Oréfice R., Clark A., West J., Brennan A., Hench L. (2007). Processing, properties, and in vitro bioactivity of polysulfone-bioactive glass composites. J. Biomed. Mater. Res. A.

[23] Wang M., Yue C.Y., Chua B. (2001). Production and evaluation of hydroxyapatite reinforced polysulfone for tissue replacement. J. Mate. Sci. Mater. Med..

[24] Ding S.J. (2007). Biodegradation behavior of chitosan/calcium phosphate composites. J. Non-Crystal. Solids.

[25] Bhat K.A., Prakash P.L., Manoharan N., Lakshmibai A., Sangeetha D. (2013). Fabrication of polymethyl methacrylate/polysulfone/nanoceramic composites for orthopedic applications. J. Appl. Polym. Sci..

[26] Babar Munir H.M., Yasin S., Iqbal T., Qamar S., Ahmad A., Mahmood H., Moniruzzaman M. (2024). Thermomechanical evaluation of zinc oxide/hydroxyapatite/high-density polyethylene hybrid composites. J. Appl. Polym. Sci..

[27] Cao J., Yang S., Liao Y., Wang Y., He J., Xiong C., Shi K., Hu X. (2023). Evaluation of polyetheretherketone composites modified by calcium silicate and carbon nanotubes for bone regeneration: Mechanical properties, biomineralization and induction of osteoblasts. Front. Bioeng. Biotechnol..

[28] Robinson P., Wilson C., Mecholsky J. (2014). Processing and mechanical properties of hydroxyapatite–polysulfone laminated composites. J. Eur. Ceram. Soc..

[29] Marcolongo M., Ducheyne P., Garino J., Schepers E. (1998). Bioactive glass fiber/polymeric composites bond to bone tissue. J. Biomed. Mater. Res..

[30] Huang S.C., Wu B.C., Ding S.J. (2015). Stem cell differentiation-induced calcium silicate cement with bacteriostatic activity. J. Mater. Chem. B.

[31] Wu I.T., Chu Y.H., Huang Y.R., Chen C.C., Ding S.J. (2022). Antibacterial ability and osteogenic activity of polyphenols-tailored calcium silicate bone cement. J. Mater. Chem. B.

[32] Wang X., Zhou Y., Xia L., Zhao C., Chen L., Yi D., Chang J., Huang L., Zheng X., Zhu H. (2015). Fabrication of nano-structured calcium silicate coatings with enhanced stability, bioactivity and osteogenic and angiogenic activity. Colloids Surf. B.

[33] Wu I.T., Kao P.F., Huang Y.R., Ding S.J. (2020). In vitro and in vivo osteogenesis of gelatin-modified calcium silicate cement with washout resistance. Mater. Sci. Eng. C.

[34] Edanami N., Takenaka S., Ibn Belal R.S., Yoshiba K., Takahara S., Yoshiba N., Ohkura N., Noiri Y. (2023). In vivo assessment of the apatite-forming ability of new-generation hydraulic calcium silicate cements using a rat subcutaneous implantation model. J. Funct. Biomater..

[35] Huang Y.R., Wu I.T., Chen C.C., Ding S.J. (2020). In vitro comparisons of microscale and nanoscale calcium silicate particles. J. Mater. Chem. B.

[36] Huang Y.H., Wu I.T., Chen C.C., Ding S.J. (2024). Synergistic effect of polyethylene glycol and lactic acid on handling properties and antibacterial efficacy of premixed calcium silicate cement. J. Funct. Biomater..

[37] Li B., Bian X., Hu W., Wang X., Li Q., Wang F., Sun M., Ma K., Zhang C., Chang J. (2020). Regenerative and protective effects of calcium silicate on senescent fibroblasts induced by high glucose. Wound Rep. Reg..

[38] Cheng W., Chang J. (2006). Fabrication and characterization of polysulfone–dicalcium silicate composite films. J. Biomater. Appl..

[39] Yudaev P.A., Chistyakov E.M. (2024). Progress in dental materials: Application of natural ingredients. Russ. Chem. Rev..

[40] Chen B., Berretta S., Evans K., Smith K., Ghita O. (2018). A primary study into graphene/polyether ether ketone (PEEK) nanocomposite for laser sintering. Appl. Surf. Sci..

[41] Bouchareb S., Doufnoune R., Riahi F., Cherif-Silini H., Belbahri L. (2021). High performance of polysulfone/graphene oxide-silver nanocomposites with excellent antibacterial capability for medical applications. Mater. Today Commun..

[42] Fiaschini N., Giuliani C., Vitali R., Tammaro L., Valerini D., Rinaldi A. (2022). Design and manufacturing of antibacterial electrospun polysulfone membranes functionalized by Ag nanocoating via magnetron sputtering. Nanomaterials.

[43] Ionita M., Pandele A.M., Crica L., Pilan L. (2014). Improving the thermal and mechanical properties of polysulfone by incorporation of graphene oxide. Compos. B.

[44] Dong X., Jeong T.J., Kline E., Banks L., Grulke E., Harris T., Escobar I.C. (2020). Eco-friendly solvents and their mixture for the fabrication of polysulfone ultrafiltration membranes: An investigation of doctor blade and slot die casting methods. J. Membr. Sci..

[45] Mehta R., Brahmbhatt H., Mukherjee M., Bhattacharya A. (2017). Tuning separation behavior of tailor-made thin film poly(piperazine-amide) composite membranes for pesticides and salts from water. Desalination.

[46] Yu L., Zhao D., Wang W. (2019). Mechanical properties and long-term durability of recycled polysulfone plastic. Waste Manag..

[47] Zambianchi M., Aluigi A., Capobianco M.L., Corticelli F., Elmi I., Zampolli S., Stante F., Bocchi L., Belosi F., Navacchia M.L. (2017). Polysulfone hollow porous granules prepared from wastesof ultrafiltration membranes as sustainable adsorbent for water and air remediation. Adv. Sustain. Syst..

[48] Khaliha S., Tunioli F., Foti L., Bianchi A., Kovtun A., Marforio T.D., Zambianchi M., Bettini C., Briñas E., Vázquez E. (2024). Upcycling of plastic membrane industrial scraps and reuse as sorbent for emerging contaminants in water. Environ. Sci. Water Res. Technol..

[49] Yaszem M.J., Paynet R.G., Hayes W.C., Lange R., Mikos A.G. (1996). Evolution of bone transplantation: Molecular, cellular and tissue strategies to engineer human bone. Biomaterials.

[50] Peroglio M., Gremillard L., Gauthier C., Chazeau L., Verrier S., Alini M., Chevalier J. (2010). Mechanical properties and cytocompatibility of poly(ε-caprolactone)-infiltrated biphasic calcium phosphate scaffolds with bimodal pore distribution. Acta Biomater..

[51] Ding S.J., Chu Y.H., Chen P.T. (2021). Mechanical biocompatibility, osteogenic activity and antibacterial efficacy of calcium silicate-zirconia biocomposites. ACS Omega.

[52] Jeyachandran P., Bontha S., Bodhak S., Balla V.K., Kundu B., Doddamani M. (2020). Mechanical behaviour of additively manufactured bioactive glass/high density polyethylene composites. J. Mech. Behav. Biomed. Mater..

[53] Wang H., Chen P., Shu Z., Chen A., Su J., Wu H., Chen Z., Yang L., Yan C., Shi Y. (2023). Laser powder bed fusion of poly-ether-ether-ketone/bioactive glass composites: Processability, mechanical properties, and bioactivity. Compos. Sci. Technol..

[54] Hu Q., Li B., Wang M., Shen J. (2004). Preparation and characterization of biodegradable chitosan/hydroxyapatite nanocomposite rods via in situ hybridization: A potential material as internal fixation of bone fracture. Biomaterials.

[55] Costa M.R., Filho J.A.C., Luna C.B.B., Dantas G.M.P., Costa A.C.F.d.M., Oliveira N.M.d.S. (2024). Toward the Production of Hydroxyapatite/Poly(Ether-Ether-Ketone) (PEEK) Biocomposites: Exploring the Physicochemical, Mechanical, Cytotoxic and Antimicrobial Properties. Polymers.

[56] Danilova S.N., Yarusova S.B., Kulchin Y.N., Zhevtun I.G., Buravlev I.Y., Okhlopkova A.A., Gordienko P.S., Subbotin E.P. (2021). UHMWPE/CaSiO_3_ Nanocomposite: Mechanical and Tribological Properties. Polymers.

[57] Senra M.R., Vieira Marques M.F., Saboya Souza D.H. (2020). Ultra-high molecular weight polyethylene bioactive composites with carbonated hydroxyapatite. J. Mech. Behav. Biomed. Mater..

[58] Kokubo T., Kim H.M., Kawashita M. (2003). Novel bioactive materials with different mechanical properties. Biomaterials.

[59] Ramakrishna S., Mayer J., Wintermantel E., Leong K.W. (2001). Biomedical applications of polymer-composite materials: A review. Compos. Sci. Technol..

[60] Peitl O., Oréfice R.L., Hench L.L., Brennan A.B. (2004). Effect of the crystallization of bioactive glass reinforcing agents on the mechanical properties of polymer composites. Mater. Sci. Eng. A.

[61] Oréfice R., West J., LaTorre G., Hench L., Brennan A. (2010). Effect of long-term in vitro testing on the properties of bioactive glass-polysulfone composites. Biomacromolecules.

[62] Moore W.R., Graves S.E., Bain G.I. (2001). Synthetic bone graft substitutes. ANZ J. Surg..

[63] Geetha M., Singh A.K., Asokamani R., Gogia A.K. (2009). Ti based biomaterials, the ultimate choice for orthopaedic implants—A review. Prog. Mater. Sci..

[64] Goodship A.E., Kenwright J. (1985). The influence of induced micromovement upon the healing of experimental tibiai fractures. J. Bone Jt. Surg..

[65] Molster A., Grjerdet N.R., Raugstad T.S., Hvidsten K., Alho A., Bang G. (1982). Effect of instability on experimental fracture healing. Acta Orthop. Scand..

[66] Mailhot J.M., Sharawy M.M., Galal M., Oldham A.M., Russell C.M. (1996). Porous polysulfone coated with platelet-derived growth factor-BB stimulates proliferation of human periodontal ligament fibroblasts. J. Periodontol..

[67] Jozwiak A., Karlik W., Wiechetek M., Werynski A. (1998). Attachment and metabolic activity of hepatocytes cultivated on selected polymeric membranes. Int. J. Artif. Organs.

[68] Fonsato V., Herrera M.B., Buttiglieri S., Gatti S., Camussi G., Tetta C. (2010). Use of a rotary bioartificial liver in the differentiation of human liver stem cells. Tissue Eng. C Methods.

[69] Stankova L., Fraczek-Szczypta A., Blazewicz M., Filova E., Blazewicz S., Lisa V., Bacakova L. (2014). Human osteoblast-like MG 63 cells on polysulfone modified with carbon nanotubes or carbon nanohorns. Carbon.

[70] Bozic K.J., Kurtz S.M., Lau E., Ong K., Chiu V., Vail T.P., Rubash H.E., Berry D.J. (2010). The epidemiology of revision total knee arthroplasty in the United States. Clin. Orthop. Relat. Res..

[71] Kapadia B.H., Berg R.A., Daley J.A., Fritz J., Bhave A., Mont M.A. (2016). Periprosthetic joint infection. Lancet.

[72] Kitridis D., Savvidis P., Cheva A., Papalois A., Givissis P., Chalidis B. (2023). Are absorbable plates more resistant to infection than titanium implants? an experimental pre-clinical trial in rabbits. J. Funct. Biomater..

[73] Liu T.M., Xu J.J., Qiu Y.R. (2017). A novel kind of polysulfone material with excellent biocompatibility modified by the sulfonated hydroxypropyl chitosan. Mater. Sci. Eng. C.

[74] Harun Z., Yusof K.N., Yunos M.Z., Shohur M.F., Jamalludin M.R. (2016). Antibacterial polysulfone membranes: The effect of eugenol and zinc oxide as additives. Mater. Sci. Forum.

[75] Zhang G., Zhou M., Xu Z., Jiang C., Shen C., Meng Q. (2019). Guanidyl-functionalized graphene/polysulfone mixed matrix ultrafiltration membrane with superior permselective, antifouling and antibacterial properties for water treatment. J. Colloid Interface Sci..

